# Coprophagy in nineteenth-century psychiatry

**DOI:** 10.1080/16512235.2018.1535737

**Published:** 2018-11-08

**Authors:** Alison M. Moore

**Affiliations:** School of Humanities and Communication Arts, Western Sydney University, Penrith, Australia

**Keywords:** Coprophilia, history of coprophilia, history of scatological behaviour, history of excrement as medicine

## Abstract

This paper shows how Austrian psychiatrists of the 1870s developed the first pathological accounts of institutional coprophagia, examining how they related the behaviour to mental illness and dementia. These ideas about coprophagia contrasted dramatically to the long European pharmacological tradition of using excrement for the treatment of a wide range of health conditions. Recent medical scholarship on institutional coprophagia is also reviewed here, with a novel hypothesis proposed about why some patients in long-term care resort to the behaviour in institutions where there is little opportunity for healthy human–microbe interactions.

Among the perverse and pathological behaviours catalogued by European psychiatrists in the second half of the nineteenth century, we find a new concern with the misuse (smearing or eating) of excrement, which came to be taken as a sign of psychopathology. This practice was now designated by several neological terms: ‘coprophagy’, ‘coprophilia’, or the German term ‘*Skatophagie’* (scatophagia) proposed by a group of Austrian psychiatrists in the 1870s who were collectively fascinated by the problem. In the first part of the paper, I consider the new nineteenth-century view of excrement that helped to produce the modern psychiatric category of psychopathological coprophagia. In the second part, I contrast these developments to the long historical tradition of what the seventeenth-century German physician Christian Paullini (Figure 3) called *Dreck Apotheke* – Filth Pharmacy [1]. Coprophagic and coprophilic behaviours among psychiatric patients attract a continuing scholarly inquiry in our own time, and a considerable body of scientific hypothesis has been suggested along the lines of an intuitive self-medicating motivation. In the final part of this paper, I review several of these hypotheses, as well as offering some additional possibilities worth investigating in light of the emerging models of the role of intestinal bacteria in regulating neurotransmitter balance, mood, and well-being. There is little indication of such a category of behaviour defined in medical sources prior to the 1870s. This is probably not because madness never produced excremental behaviours of this kind. In fact, the early-modern Dutch physician Jan Baptise Van Helmont described a painter in Brussels who had gone mad and thereafter ate his own excrement [2]. The question then is why did this only result in a meaningful medical category of behaviour from the late nineteenth century onwards?

**Figure 1.**
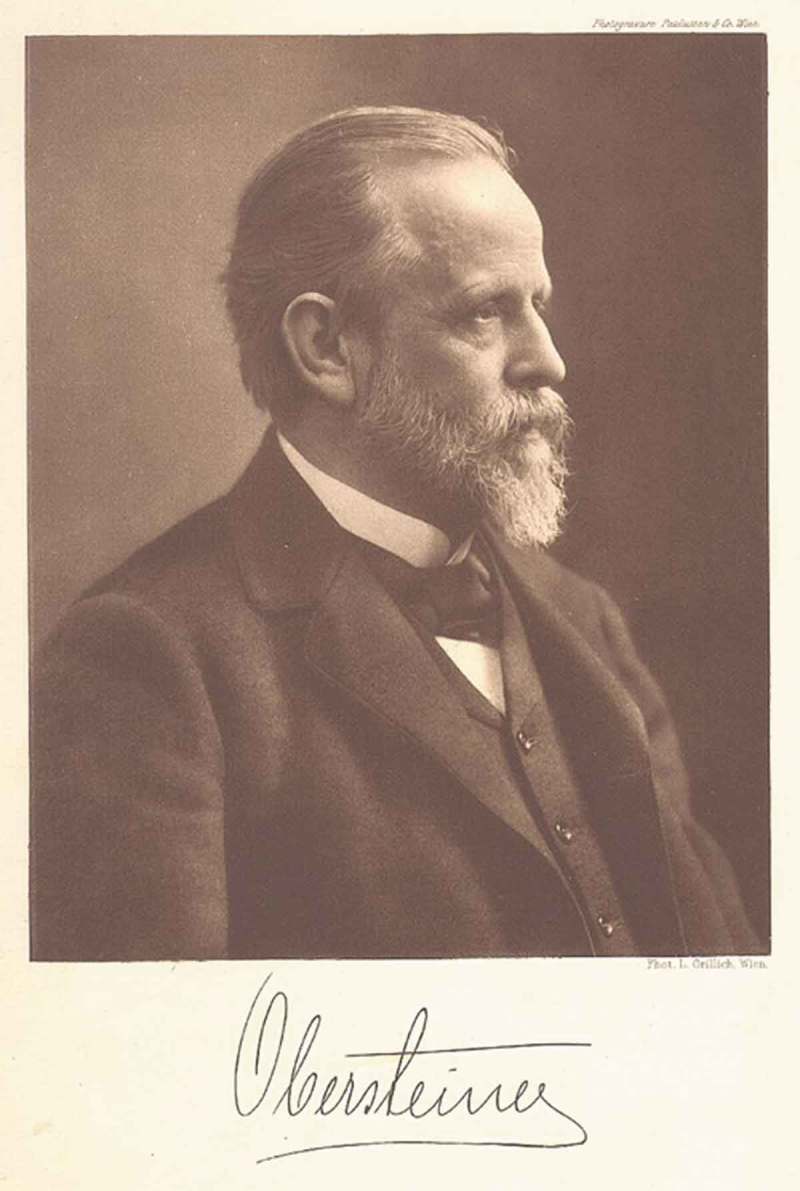
A signed photograph of the Viennese psychiatrist Heinrich Obersteiner taken around 1900. Wikimedia Public Domain.

**Figure 2.**
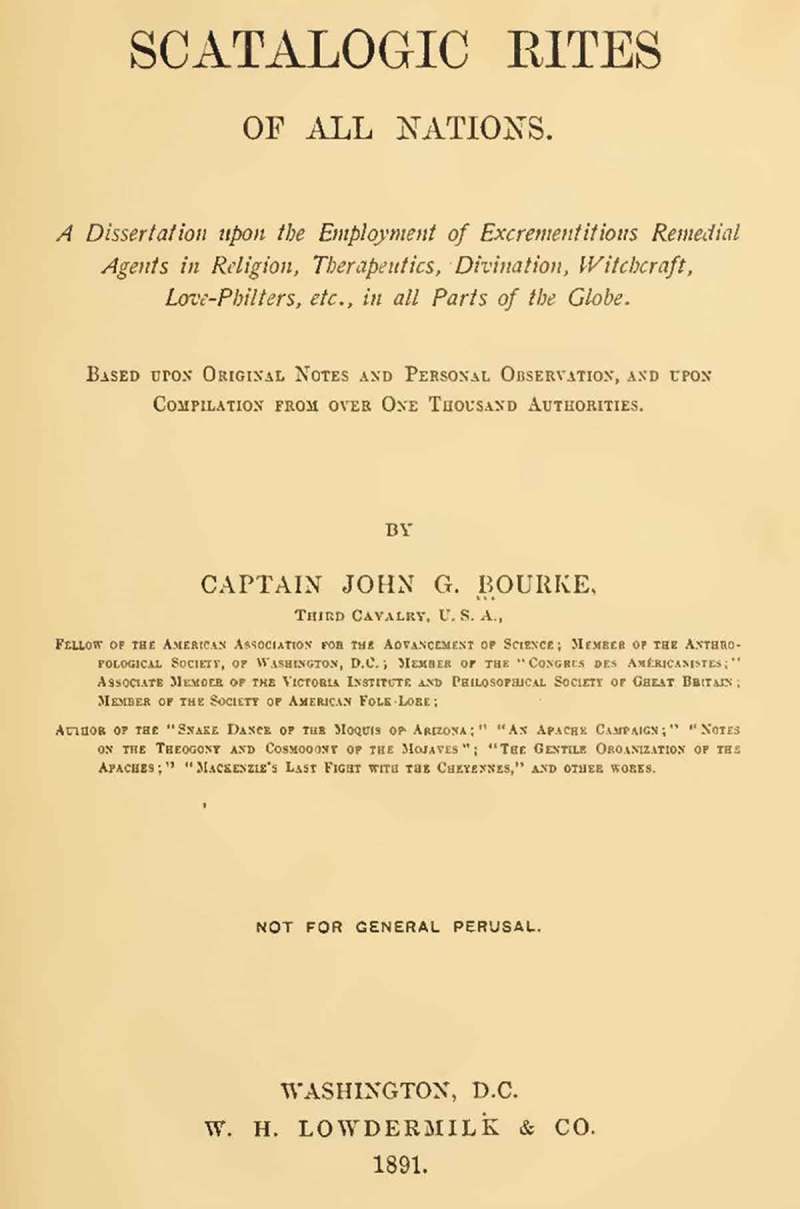
The title page of the 1891 edition of John G. Bourke’s compendium. Courtesy of Archive.org. Public Domain.

**Figure 3.**
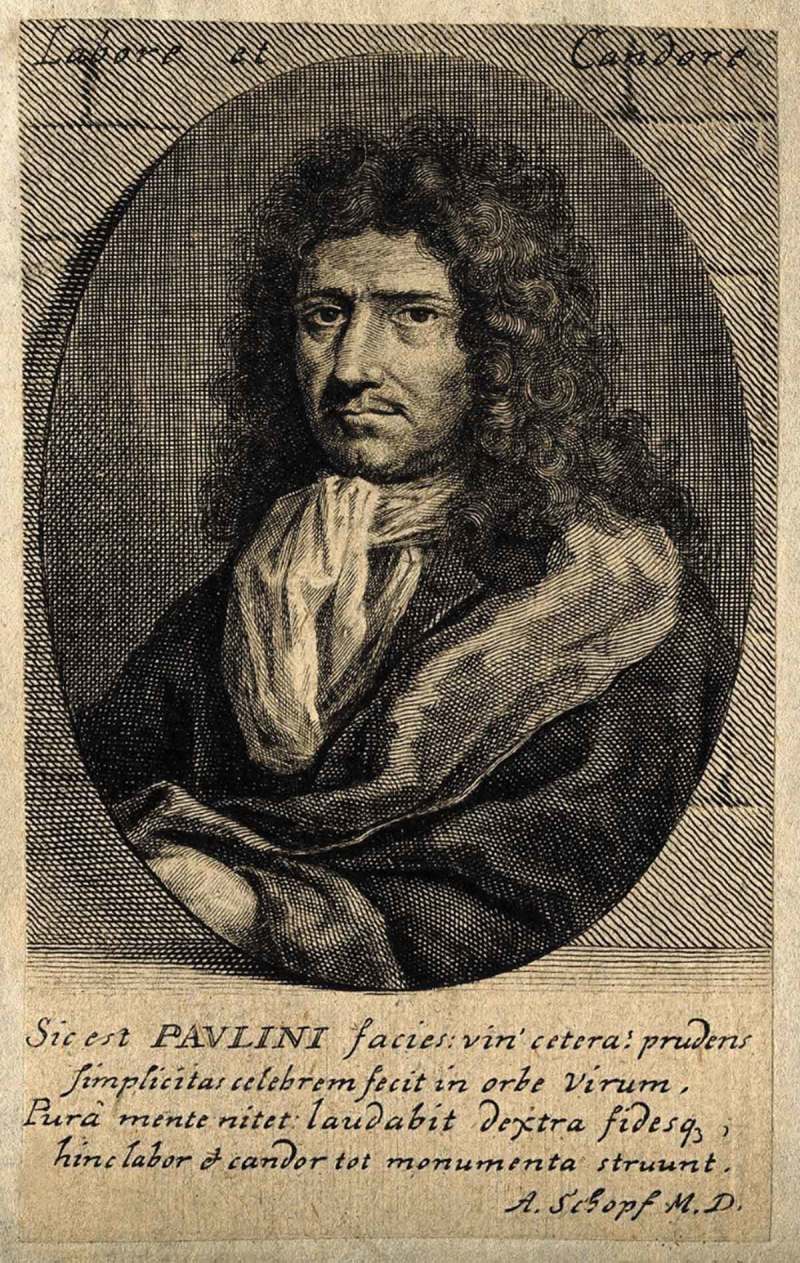
Portrait of the early-modern German physician Franz Christian Paullini, author of *Heilsame Dreck Apotheke* (*Therapeutic Filth Pharmacy*) of 1696. Courtesy of the Wellcome Collection.

The answer proposed here is that from the end of the nineteenth century, European medical understanding entered a radically new period defined by an important rupture in ideas about the meaning of excrement. From the time of the ancient Egyptians (circa 1550 BCE) until the late eighteenth century, faecal remedies had been common in European pharmacology, with excrements of different animals, including humans, blended with other agents to produce medications that featured in all the standard apothecary lists [3]. There was simply no place to consider the eating of excrement as, per se, a sign of madness in a context in which excrement was widely agreed to have a pharmacological value. Nineteenth-century doctors were certainly aware of these long and ancient traditions, but with help of new ethnographic colonial imaginaries and their uptake in the theorisation of Freudian psychoanalysis, and with the help of the new fashion for theories of intestinal autointoxication, a reframing of their meaning occurred: Early-modern excremental pharmacology was now simply viewed as the remnant of primitive cultures that had failed to differentiate muck from what mattered. They were of no interest to doctors in the new scientific era of the nineteenth century, and indeed the eating of excrement could now only be taken as a sign of civilisation’s discontents: the insane.

## Austrian psychiatric ideas about coprophagia before Freud

By the time the first psychiatrists working in insane asylums began to observe the coprophagic behaviour of a small number of patients, they were so distanced from the notion of excrement having any potential pharmacological value, that this possibility of behavioural explanation did not even appear to occur to them. Instead, they worried that the eating of excrement might itself cause mental illness. These psychiatrists included Professor Lang who was director of the *Landes-Irrenanstalt* (Lunatic Asylum) in Graz; the senior German psychiatrist Adolf Albrecht Erlenmeyer, who authored a major work on syphilitic psychosis [4]; Heinrich Obersteiner (Figure 1), a reputable Jewish psychiatrist in whose Vienna clinic the young Sigmund Freud had worked around 1888, following his apprenticeship in Paris under Jean-Marie Charcot in the early 1880s [5]; and a Dr Maresch who was editor in chief of *Psychiatrisches Centralblatt*, a new Austrian medical journal founded in 1871, in which all the others named here published articles on coprophagia.

Because of Freud’s connection with Obersteiner, it is worth inquiring if the ideas about coprophagia generated by this group of Austrian psychiatrists formed part of the genealogy of the Freudian concepts of defecatory sublimation in childhood as necessary for adult psycho-development. Freud, among all the psychoanalytic and psychiatric thinkers of the late nineteenth and early twentieth centuries, particularly privileged defecation in psychosexual development, positioning the anal-sadistic phase as the most primitive instinctive moment of struggle in the development of the child psyche in modern civilisation. His ideas about this, as I have previously described, were deeply idiosyncratic and profoundly teleological in situating defecatory behaviour within a vision of ‘recapitulation’ of the child through earlier stages of civilisational development [6]. I have, in earlier work, shown how Freud’s ideas about anal primitivity engaged with late-nineteenth-century ethnographic observations about excremental practices in diverse cultures, particularly the 1888 *Compilation of Notes and Memoranda Bearing Upon the Use of Human Ordure and Human Urine in Rites of a Religious or Semi-Religious Character Among Various Nations* by the US cavalryman John G. Bourke [7] (Figure 2). The current paper considers the earlier body of Austrian psychiatric thought on the relationship between coprophagia and mental illness which most likely also helped Freud’s ideas about excrement and primitivity to cohere.

Freud himself does not appear to have enga-ged explicitly with the work of Lang, Maresch, Erlenmeyer, and Obersteiner in relation to excremental questions, though it seems likely that he would have been exposed to their ideas as an intern in Obersteiner’s clinic. He also most certainly read Bourke, who in turn cited Obersteiner [7]. Freud appears to have read some scholarship on the notion of scatological behaviour as sign of psychopathology: His 1917 paper ‘On Transformation of Instinct as Exemplified in Anal Erotism’ referred to ‘obsessional neurotics’ in whom ‘regressive debasement’ towards faeces was observed, though without citing his source for this observation [8]. In 1912, the American Freudian psychoanalyst Ernest Jones had signalled a direct genealogical link between Freudian ideas of excrement as a mark of primitivity and the earlier Austrian psychiatric scholarship on coprophagia as a form of mental illness. Jones wrote: ‘That it is not very rare for insane persons to eat their own excrement is of course well known’, footnoting Obersteiner’s data cited in the 1871 article in the *Psychiatrisches Centralblatt* which referred to the figure of 1% of patients exhibiting the behaviour [9]. Prior to this passage in the same text, Jones had cited the work of John G. Bourke on the practice of excremental rituals and remedies in ‘primitive’ cultures, adding a digression on the matter of

the association between food as taken into the body and food as it is given out, two ideas which are by no means so remote from each other in the primitive mind, including that of the child, as they usually are in that of the civilised adult. [9]

For Freud and for Jones, the psychiatric patient displaying coprophagic tendencies was regressing to childhood, with childhood itself representing a recapitulation of earlier ‘primitive’ social-evolutionary stages. The insane, toddlers, and primitive humans all shared a coprophagic disposition.

Having established with some degree of likelihood that Freud was influenced by the earlier Austrian psychiatric literature on coprophagia and mental illness, we might then inquire if these earlier discussions had viewed the behaviour as a sort of regression to childhood, or as a primitive practice in the mode of later Freudian thought. But at no point did Lang, Erlenmeyer, Maresch, or Obersteiner appear to entertain such ideas. This was clearly Freud’s original line of reasoning about the meaning of coprophagy and coprophilia. In 1896, he had written to his mentor Wilhelm Fleiss asking, ‘in connection with the eating of excrement’ if there was ever a phase in a child’s development when disgust in such things was not yet developed, adding that ‘the answer would be of theoretical interest’ [10]. He clearly found his answer to this question, as indicated in later statements to the effect that ‘the excreta arouse no disgust in children … and seem valuable to them as being part of their own body which has come away from it.’ [11]. As psychologist Nick Haslam notes, the lack of early childhood disgust towards excrement was verified in the 1986 study of Rozin et al. in which most of the 2-year old test-subjects, when offered what appeared to be a dog turd on a plate (actually a simulacra made of smelly cheese and peanut butter), voluntary put it in their mouths [12,13].

For Freud, the disgust towards excrement and the culturally appropriate abjection of it were products of the first and second phases of erotogenic sublimation in childhood that later encompassed perverse and incestuous sexual desires – the various oral, anal, and genital phases [6]. His observation of coprophagic or scatologic behaviour in adults then situated it as a form of regression or infantilisation. This idea was exciting for Freud because it fitted his emergent vision of childhood development as evolutionary recapitulation, in which infantile drives had to be overcome in the individual in the same way that primitive humans were thought to evolve towards civilisation [14]. This was a kind of cultural application of Ernest Haeckel’s notion of biological developmental recapitulation in which the human embryo passes through previous stages of animal evolution, developing pharyngeal gill slits and a post-anal tail in the eighth week of gestation. Freud thought that the civilised child in the development to adulthood had to learn to sublimate excrement just as primitive humans of the European past must once have done [6]. Adult neurosis was a regression to those infantile/primitive drives.

It was in the decade prior Freud’s work under Obersteiner when the latter was most engaged, along with Lang, Maresch, and Erlenmeyer, with the problem of aberrant excremental behaviours among inhabitants of insane asylums. They all agreed that it was not a common problem exactly, though clearly nonetheless a disturbing one for asylum medical staff and for other patients. One of the problems that these early psychiatrists faced in defining their object was the diversity of types of individuals who were ‘scatophagic’ – ranging from those with severe delusional illnesses, to those with a conscious sexual fetish for excrement (such as that described by the Marquis de Sade). That distinction probably made less sense to nineteenth-century psychiatrists than it might today since sexual perversions at this time were widely considered to constitute a form of psychopathology and were seen as signs of genetic ‘taints’, according to the thesis of ‘degeneration’ [15]. But the Austrian psychiatrists’ case studies all appeared to concern those who had never exhibited any such desires before but who at a certain age – and in institutional contexts – developed behaviours of eating or smearing their own or other patients’ excrement.

The first inspiration for the debate about *Skatophagie* appears to have been an oral paper delivered in Graz by Professor Lang in 1871, entitled ‘Über Skatophagie bei Irren’ (On Scatophagia in Madmen), which appeared in written form in the first volume the *Psychiatrisches Centralblatt* of 1872 [16]. Lang presented several case studies of scatologic patients, which included both a 26-year-old army cadet who was clearly delusional and insisted on using his own excrement as a sort of clay from which he modelled furniture for his room. Another was an educated and intelligent alcoholic man in his fifties who suffered brain damage from a fall (hitting his head) while drunk, and thereafter developed coprophagic behaviour along with other drastic changes to his personality [16]. Lang considered the eating of excrement potentially very damaging to his patients’ physiology and considered that it might even have been part of the causation of derangement, or at least part of the reason for the men’s mental deterioration over time. Was coprophagia merely an inconvenient symptom of madness that institutional staff had to manage? He doubted this, considering it might play a more sinister causative role, worthy of scientific investigation.

Erlenmeyer made a response to this paper in the *Psychiatrisches Centralblatt* of 1873, in which he repeated Obersteiner’s reported statistical account of the prevalence of coprophilic patients in asylums – 1 in 100 patients most of whom were male – and insisted that, in his own experience, it was not a masturbatory behaviour, and nor could any ‘injurious influence of the diet’ be seen [17]. In this same volume, a longer article by the journal’s editor-in-chief, doctor Maresch summarised a discussion of psychiatrists in a meeting on the matter, which included Maresch himself, Lang, Obersteiner, as well as Professor Beer and doctors Flechner and Leidesdorf. Here, Maresch noted Lang’s observation that those with less education were more likely to exhibit the behaviour and added that it was most common among those in ‘chronic maniacal states’ and in those whose mental deterioration had descended to the expression of complete nonsense. Maresch claimed that the application of a ‘constant current’ of electrotherapy effectively ceased the behaviour (perhaps along with many other behaviours!) [18]. Though it is worth noting that would have most likely been a weak current as per the customary use of electricity in late-nineteenth-century psychiatry.

But Maresch’s summary of his own and his colleagues thinking on the matter claimed that sexual fetishists indeed constituted a large percentage of those exhibiting coprophilic behaviour, and contra Erlenmeyer, insisted on it as primarily a masturbatory activity, as evidenced by the observed enthusiasm and enjoyment that coprophages showed when observed consuming excrement, both their own and that of other patients. The ingestion of the fecal matter must surely be damaging too he insisted, since the brain requires the right amounts of nutrients to function, and with excrement making up a large part of the diet of coprophagic patients, their blood must surely be improperly constituted: ‘the defective metabolism thus produced alters all functions, and causes all sorts of ruin to organic life’. By way of illustration, he described a depressed and anxious patient who had resorted to eating his own excrement in the apparent desperation to become well again but had shown a marked deterioration into a more severe form of mental derangement after adopting this unusual diet, and thereafter became permanently coprophagic, believing that it was the only food that might fuel his recovery. Consequently, he considered ‘scatophagia to be one of the most pernicious disease states … because of its highly injurious effect’ caused by ‘the production of certain agents added to the blood’, such that ‘the activities of organic life are incessantly prepared of an inappropriate admixture’. [18] By way of support, he cited an essay by the early-nineteenth-century alienist Carl Ideler entitled *‘Verbrechen und Wahnsinn’* (Crime and Insanity), in which the latter attributed ‘the mood of melancholy patients to the hydrogen sulphide gases which have developed from stagnant excremental substances that have passed into the blood’ [19].

## The nineteenth-century rupture with historical ‘Filth Pharmacy’

The Austrian psychiatrists’ insistence on the nefarious effects of excrement-eating represented an important rupture in medical thought viewed over long historical perspective. Most commonly, early-modern medical texts, in fact, referred to it as a variously useful pharmacological remedy. Several important and much-cited works of early-modern pharmacy include extensive discussion of the use of excremental remedies to be ingested orally or applied topically for the treatment of many diseases, including Johan David Ruland’s *Pharmacopoea Nova* of 1644 [20], Michael Etmüller’s *Opera Omnia* of 1690 [21], Franz Christian Paullini’s *Dreck Apotheke* (*Filth Pharmacy*) of 1696 [1], and Martin Schurig’s *Chylologia* of 1725 [22], all which, as of 2018, remain untranslated into any modern languages. In some cases, the idea of excrement as a pharmacological remedy appeared as a form of critique of irrational remedies of other kinds. For instance, the early-eighteenth-century natural philosopher Robert Boyle had noted sceptically that ‘a despised common sample, nay an infect or an excrement may in some cases prove nobler medicines than an extract, elixir, or a quintessence’ [23]. But he also prescribed ‘Paracelsus’ *zebethum occidentale*, (viz. human dung) of a good colour and consistence’ be used as a dried powder, blown into the eyes of one suffering blurry vision [24]. Though, it was important, Boyle had noted, not to use the excrements of the mad for any remedy, lest one become mad oneself [25]. Such a remark indeed may now be seen as a remarkable intuition of current scientific models of the effect of intestinal bacteria on mental health, as will be discussed in the last section of this paper.

This is not to say that all premodern views of excrement unequivocally celebrated its value. As the work of numerous medieval and early-modern literary scholars has shown, excrement came to be associated with devil, with humiliation and urban disorder in a range of texts from the fifteenth to eighteenth centuries [26–28]. Early-modern excremental medical remedies were certainly not without critics in their own time too. The English physician Nicholas Culpeper’s *Pharmacopoeia Londinensis* (London Dispensatory) of 1652 mocked the fact that the College of Physicians ‘give the apothecaries a catalogue of what part of living creatures and excrements they must keep in their shops’ [29]. In all cases though, early-modern texts certainly make no mention of coprophagic behaviour as a sign of mental illness.

**Figure 4.**
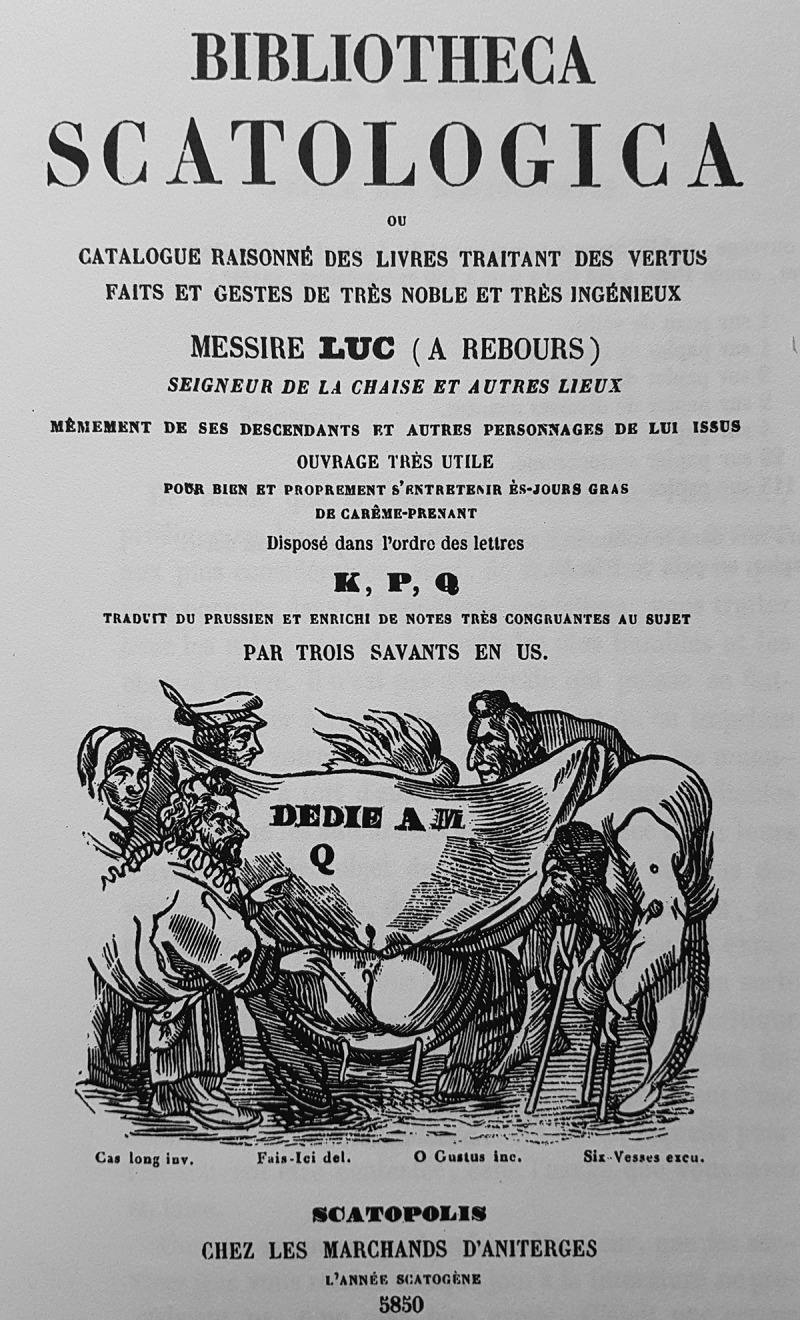
The title page of the 1849 *Bibliotheca scatologica* by Veinnant, Jannet and Payen. Author’s own photograph.

The ancient to early-modern excremental pharmacy traditions were clearly known to many doctors and psychiatrists in the nineteenth century through anthologies such as that of John G. Bourke as well as an earlier French work of 1849 entitled *Bibliotheca Scatologica*, by Auguste Veinant, Pierre Jannet, and Jean-François Payen which described the works of Schurig and Paullini in some detail [30]. Other similar bibliographies included the *Anthologie scatologique* by Pierre-Gustave Brunet of 1861 [31], and the *Bibliographie* Des *ouvrages relatifs* À *l’amour, aux femmes, au marriage et* Des *livres facétieux, scatologiques satyriques, etc. *… (Bibliography of works about love, women, marriage and facetious, scatological and satirical books, etc.…) by the editor and socialist Jules Gay, first self-published in 1861 and reprinted in several editions throughout last decades of the nineteenth century [32]. These works represented a curious intermediary stage in the divide between early-modern uses of excrement as a pharmacological agent, and the later nineteenth-century theories of coprophagia as psychopathological, infantile, or primitive. They combined scatological humour with a sort of titillated curiosity in the early-modern medical practices, jocularly naming the physicians who prescribed *stercora* (manure) ‘stercoral doctors’. They found a utility in celebrating the filth-medicine tradition, enlisting it as an ally in their atheistic critique of benevolent Christian views they claimed denied the reality of unseemly things [30]. The *Bibliotheca Scatologica*’s first edition listed its publication details as: ‘Scatopolis (Paris): *chez* les marchands d’aniterges, l’année scatogène 5850 [i.e. 1849]’: ‘Scatopolis (Paris): by the toilet-paper merchants, in the scatogenic year 5850 [i.e. 1849]’ [30] (Figure 4). Their works listed flatulence verses and scatological jokes alongside serious medical texts of the past detailing the use of excremental remedies. However, these works belonged to a quite peculiarly French context ideological opposition of atheist materialism towards Catholic faith. It is quite possible that the Austrian group of psychiatrists would have been ignorant of these works in French, and it does not appear that any similar anthologies were published on this topic in German during the nineteenth century. Both Freud and Jones certainly knew of them via John G. Bourke’s citations, but there is no evidence that they followed-up in examining the texts to which Bourke himself referred, nor is it clear how much of the early-modern medical texts Bourke himself actually read with his at-best rudimentary school-boy Latin.

We might expect to find that the major cause of the shift in the 1870s discussion of coprophagia would refer to the new bacteriological model of disease that had begun to displace the miasma model of Galenic medicine in the period between 1850s and the 1880s, following the work of Louis Pasteur in the 1850s, as well as the 1849 essay by John Snow *On the Mode of Communication of Cholera* which made a substantive case for the view of this highly prevalent disease as faecally transmitted [33]. But surprisingly, the Austrian doctors writing in the *Psychiatrisches Centralblatt* made no mention of any concerns about infection and located the negative effects of excrement-eating in a far more hygiene-based model of disease as the product of improper diet – hygiene understood here in the nineteenth-century sense of the term, as described by James C. Whorton – referring to the management of the body through diet and bodily functions [34]. Germ theory was clearly not the cause of the novel Austrian psychiatric pathologisation of coprophagia. It seems the mechanism by which they considered coprophagia to aggravate mental illness was via a notion that became popular in nineteenth-century medical thought and in quack remedies for constipation: autointoxication [34]. The Austrian psychiatrists did not use this exact term, but they did appear to be gesturing towards a similar idea: that excrement itself could poison the blood and consequently derange the mind. The idea had been current throughout German-speaking Europe, as well as in France, from the mid-nineteenth century until the 1920s, and is most associated with the work of the French pathologist Charles Jacques Bouchard [35]. Much of the most significant work on autointoxication occurred after the period in which the Austrian psychiatrists were writing on coprophagia. But the notion was clearly circulating in their time as well: From 1868, the Prussian physician Hermann Senator had referred to the role of intestinal putrefaction and the development of diseases, using the term *Selbstinfection* (self-infection) [36]. In his later work, he theorised about it as the cause of delirium [37]. The Berlin physician Ludwig Brieger’s work on autointoxication in the 1880s made an explicit connection between intestinal microbes (specifically anaerobes) and the generation of toxic by-products, but earlier theories of autointoxication on which much of the late-nineteenth-century fixation with enemas rested referred only vaguely to the ‘putrefaction’ that faecal retention was thought to generate [38]. Excrement itself was already considered poisonous in the mid-nineteenth-century medical imaginary, and germ theory merely served to provide a further layer of mechanistic explanation.

The *Psychiatrisches Centralblatt* writings on *Skatophagie* appear to have remained fairly obscure – they are not cited, for instance, by the great Austrian psychiatrist Richard von Krafft-Ebing in his description of excremental sexual fantasies in the *Psychopathia Sexualis* of 1886. Krafft-Ebing’s ‘corprolagnic’ case studies all refer to erotic fetishes of high-functioning individuals in which the defilement with excrement or ingestion of it featured as a dramatised act of sexual submission and humiliation, e.g. cases 79, 80, 82 [39]. However, it seems likely that the 1870s accounts of coprophagic asylum patients as masturbatory in their enjoyment helped to produce the view of it as primarily a form of sexual perversion in the account of Kraftt-Ebing and others in the 1880s and 1890s. That view, in turn, was probably also a stimulus for the later Freudian account of childhood coprophilia as a key component of psycho-sexual development.

## Institutional coprophagia today

**Figure 5.**
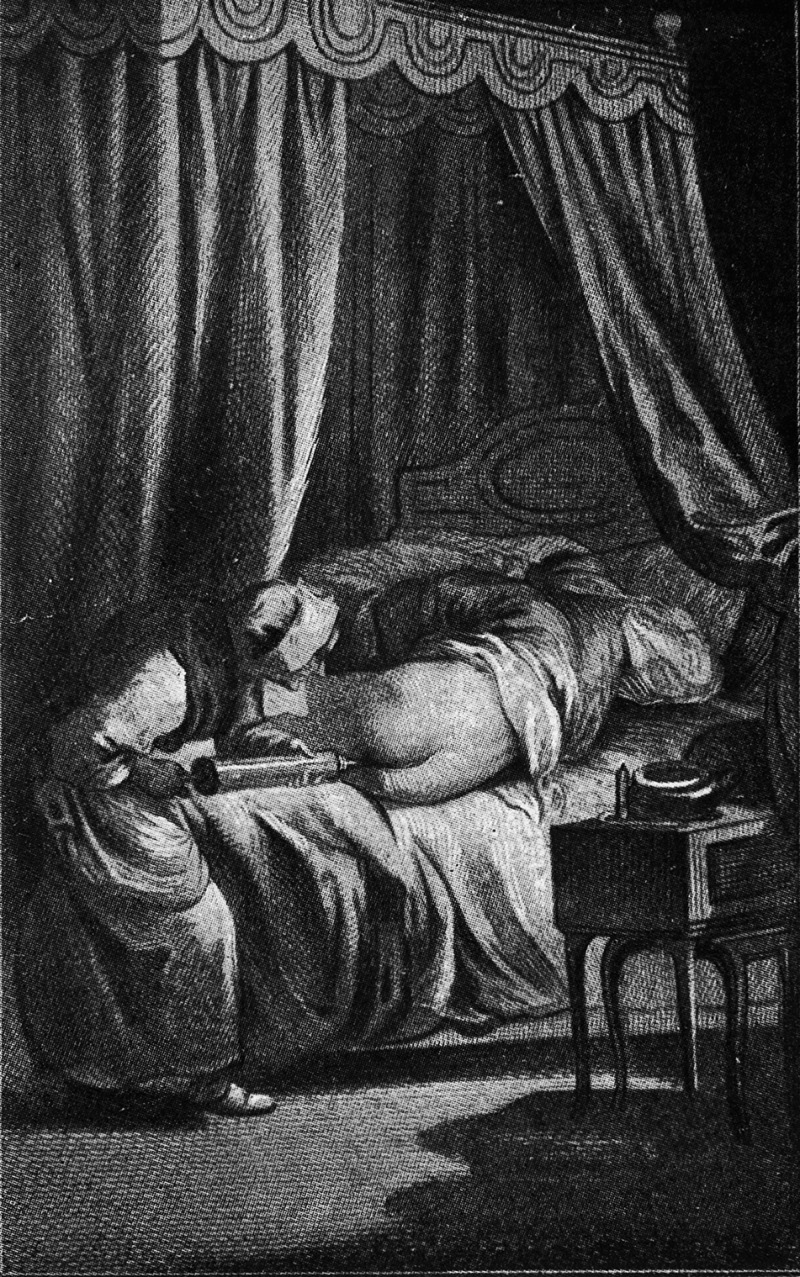
A French drawing of a nurse administering an enema to a bed-ridden patient, circa 1800. Courtesy of the Wellcome Collection.

Since the 1980s, there has again developed a clinical literature on coprophagia in varied patient populations, including children with gastrointestinal problems, children and adults with mental handicaps, elderly adults with advanced dementia, and adults with dissociative psychoses, beginning with the 1987 paper by the two psychiatrists Nissan and Haggag, which described episodic coprophagia in a female sufferer of Major Affective Disorder (DMS-III bipolar mixed type), and hypothesised a ‘reversion of the normal process whereby experience and ideation give rise to affect’ in the amygdala [40]. A number of clinicians between 1989 and 2017 published case reports, some indicating anecdotal success in reducing coprophagic and scatologic incidents through the use of various drugs and behavioural protocols in adults and children with mental handicaps [41], in a schizophrenic adult [42], and in children brought to a gastroenterology clinic because of constipation and encopresis [43]. Other studies have hypothesised about the causes of scatological behaviours in relation to obsessive–compulsive disorder [44], in relation to dementia [45], in the geriatric mentally ill [46], in relation to developmental handicaps [47], and as sexual fantasy reported by patients in psychotherapy [48]. A 2016 study by researchers at the Mayo Clinic falsely asserted the earliest publication on coprophilia in mental asylums was that of Theodor Kellogg in 1897, in a medical textbook written some 16 years after the Austrian scholarship identified in the current paper [49]. Kellogg’s brief mention appears to be the first in the English language, after which a long hiatus is probably explained by the solution Kellogg indicated to be widely used in US asylums at the turn of the century and most probably throughout the twentieth century as well: compulsory, repeated administration of enemas so that such patients never had anything to play with! [50] (Figure 5)

As the authors of a 2016 Greek study on the problem note, coprophagic behaviour in institutional settings causes significant loss of quality of life for patients who display it as it tends to result in them being isolated in special wards, avoided by nursing staff and other patients, and can result in physical restraint and severe limitations of freedom of movement in the attempt to prevent them engaging in the behaviour [51]. Gerontology researcher Joan Ostaszkiewicz has suggested that urinary and faecal incontinence alone may be a risk-factor for elder abuse and can be subject to chastisement and shaming on the part of some carers [52]. Some current scholarship clearly too carries the legacy of psychoanalytic thinking about coprophilia as evidenced in the remarks about the behaviour representing a regression to infancy or to ‘primitive, primordial instincts’ found in certain publications [51]. It is possible that coprophagic patients in many cases are not being respectfully cared for and are harshly judged by institutional staff on account of the powerful conditioning of disgust towards excrement that has become generalised in modern cultures.

On the other hand, the use of excrement as a legitimate therapeutic remedy has returned in modern medicine in the form of faecal microbial transplant for *Clostridium difficile* infection, at an efficacy rate that far exceeds competing antibiotic remedies [53,54]. It also shows promise as a treatment for persistent Crohn’s disease and ulcerative colitis [55,56]. When we consider this alongside the recognition that throughout the history of medicine, there have been uses of excrement as a pharmacological remedy for various conditions, it is most certainly worth considering whether institutional forms of coprophagia may be caused by an intuitive self-medicating motivation. It is now known that a wide variety of animals display zoopharmacognosy, or the ability to intuitively self-medicate, either by learnt behaviours in intelligent primates (such as the chimpanzee use of antiparasitic herbs), or through innate adaptive mechanisms and without the need for high intelligence, explaining its occurrence in ants, moths, and fruit flies [57–59]. Some researchers have indeed considered a potential self-medicating explanation for human coprophagia, noting its use by different animals (rabbits, gorillas) to meet nutritional deficiencies such as for the B vitamin thiamine [60]. However, no consistent vitamin or mineral deficiencies have been identified in human excrement-eaters to date. On the other hand, one study found success in reducing coprophagic incidents in a man with profound retardation and autism through the provision of highly spiced foods *ad libidum* [61].

Since current research on institutional coprophagia has already approached it through the rubric of possible self-medication approaches, it is surprising that none of these studies have considered that coprophagia may, in some instances, be motivated by an intuitive quest for commensal intestinal microbes. Clinicians dealing with this challenge may wish to consider the growing evidence of the importance microbial ecology in human mental and general health, particularly in relation to microbes that: (a) generate the neuroprotective short-chain fatty acids n-butyrate, acetate, and propionate as by-products of their own metabolism [62]; (b) synthesise Menaquinones (vitamin K2) which play an important role in bone remineralisation and calcium regulation – of particular relevance to osteopenia in the elderly [63]; (c) produce indoles such as indolamine-2,3-dioxygenase, which act as catalysing enzymes in tryptophan synthesis, with corresponding beneficial effects on the gut epithelium, but also on serotonin synthesis [64,65]. They might also wish to consider the bacterial species that have been found to upregulate neurotransmitters and neurotransmitter precursors, including GABA, Dopamine, 5HT, and acetylcholine – of particular relevance to mental illness and to neurodegenerative diseases such as Alzheimer’s and Parkinson’s [66].

There is now a considerable body of scientific evidence of the role of intestinal microbiota in regulating a wide range of animal physiological functions, and an emerging paradigmatic shift towards a view of it in humans as an organ of the body that has co-evolved with us from our earliest multicellular beginnings – as much a part of ‘us’ as the microbes from which our own cellular mitochondria are thought to have evolved [67–69]. The evidence accumulating of a role of the intestinal microbiota in mental health, in particular, might then prove of interest to carers both of the mentally ill and of those suffering depression or neurodegenerative diseases in aged care towards a revision of the nutritional and ecological environment provided to those in institutional and in-home settings [70–73].

There is evidence from a number of difference cultures indicating that diets provided to inhabitants of long-term care facilities tend to be low in fermentable polysaccharides of the kind that would promote the growth of commensal intestinal bacteria [74–76]. Considering the long history of the use of excremental remedies in the medical traditions of numerous human cultures, it would seem a fruitful line of inquiry to consider whether self-medicating drives towards coprophagia might be located in the abundance of microbiota present in excrement, which are, otherwise, lacking in the intestinal tracts of those in institutionalised medical settings. Long-term care institutions, whether nursing homes for the elderly, care facilities for the mentally handicapped, or psychiatric hospitals, are all frequently (though not ubiquitously) characterised by a lack of microbial–ecological consideration in meal planning, with the need for greater nutritional variety that includes both copious prebiotic (soluble) fibre in the form of fresh vegetables and pulses, as well as probiotic foods (such as fermented vegetables, grains, and dairy products). Many institutions frequently disinfect tactile interior surfaces, keep residents indoors most, if not all, of the time, without access to pets, without physical intimacy with other humans, and with limited opportunity to make physical contact with the natural environment – all of which are important sources of microbial inoculation in humans. It would seem worth experimenting in clinical settings to see if coprophagic patients fed a diet aimed at creating a more diverse and robust intestinal microbiome, which includes palatable probiotic and prebiotic foods given *ad libidum*, and permitted access to gardening, pets, or lying on grass lawns might be less inclined to seek microbial support from faeces.
